# Enzymatic catalysis for sustainable production of high omega‐3 triglyceride oil using imidazolium‐based ionic liquids

**DOI:** 10.1002/fsn3.733

**Published:** 2018-08-06

**Authors:** Hong Fu, Mengqi Li, Ruimin Ni, Yangming Martin Lo

**Affiliations:** ^1^ College of Biological Science and Engineering Fuzhou University Fuzhou, Fujian China; ^2^ Fujian Provincial Key Laboratory of Marine Enzyme Engineering Fuzhou University Fuzhou, Fujian China

## Abstract

Two different fish oil preparations, namely triglycerides and ethyl esters containing, respectively, 30.02% and 74.38% of omega‐3 fatty acids, were employed as the substrates for transesterification. Catalyzed by immobilized lipase using imidazolium‐based ionic liquid systems, the total content of eicosapentaenoic acid (EPA) and docosahexaenoic acid (DHA) in the resulting triglyceride reached 63.60% when 4% hydrophobic ionic liquid was used, which was 11.74% higher than that of the triglyceride produced in a solvent‐free reaction system. The activation energy of the product (triglyceride‐type fish oil) was 173.64 KJ mol^−1^, which was not significantly different from that of the commercial ethyl ester‐type fish oil, so were the other thermal oxidative kinetic parameters. The kinetic parameters depicting the thermal and oxidative stability of the fish oil product provide the basis for industrial processing, storage, and applications.

## 1 INTRODUCTION

Fish oil products containing elevated contents of omega‐3 polyunsaturated fatty acids (n‐3 PUFA) has for decades been the focus of the fish oil industry owing to their recognized benefits such as reducing the risk of heart disease and inflammatory disease and ameliorating brain function and mental health (Cheong, Guo, Yang, Chua, & Xu, 2011; Ruxton, Reed, Simpson, & Millington, 2004). Recently, additional benefits continued to be reported, including promising applications in the management of gastrointestinal cancer (Eltweri et al., 2017), the ability to ameliorate high‐fructose diet‐induced hepatic steatosis and hyperlipidemia (Oishi et al., 2018), beneficial for immunoregulation, and suppressing atherosclerosis development (Subash‐Babu & Alshatwi, 2018). However, many conventional chemical methods for the production of marine oils containing n‐3 PUFA involve the usage of water‐immiscible organic solvents that are toxic, flammable, or volatile, subsequently raising the overall production cost due to the need to properly dispose of the residual organic solvents (Ackman, Ratnayake, & Olson, 1988; Halldorsson, Kristinsson, & Haraldsson, 2004; Teramoto, Matsuyama, Ohnishi, Uwagawa, & Nakai, 1994; Yokochi, Usita, Kamisaka, Nakahara, & Suzuki, 1990).

Ionic liquids (ILs), ion containing liquids, due to their negligible vapor pressure and excellent thermal stabilities, are considered promising “green” and “designer” media for the extraction and separation of bioactive compounds from diverse origins over a large temperature range, from below ambient to well over 300–400°C (Erbeldinger, Mesiano, & Russell, 2000; Nowicki & Muszynski, 2014; Sivapragasam, Moniruzzaman, & Goto, 2016; Ventura et al., 2017; Wei, Yang, & Chen, 2003; Zhao, Xia, & Ma, 2005). Comprised of a bulky asymmetric cation in combination with any of a wide variety of anions, ILs as organic salts that exist as liquids at a low temperature (<100°C) thus cannot be packed easily, making them designable for polarity and hydrophobicity (Baudequin et al., 2003; Brennecke & Maginn, 2001; Tao, Dong, Pavlidis, Chen, & Tan, 2016), and capable of inhibiting crystallization (Cheong et al., 2011; Zhao et al., 2005). Therefore, ILs are deemed a new alternative for enzyme‐catalyzed synthesis in environmentally friendly environments as solvents, cosolvents, cosurfactants, electrolytes, and adjuvants (Cooney & Benjamin, 2016; He, Li, Liu, Li, & Liu, 2005; Lue, Guo, & Xu, 2007; Pei, Wang, Wu, Xuan, & Lu, 2009; Zhu, Wang, Wang, Li, & Wang, 2009). Since the structures of cations and anions are crucial in making ILs hydrophilic or hydrophobic, structure‐wise, the anion portion has been found to be more crucial in determining the water miscibility of ILs (Huddleston et al., 2001). The ILs based on [PF_6_]^−^ (hexafluorophosphate) and [Tf_2_N]^−^ {= bis[(trifluoromethyl)sulfonyl]amide} are normally water‐immiscible, making them the solvents of choice for forming biphasic systems in most IL applications (Zhao et al., 2005).

Moreover, the pioneer work on recovery of n‐3 PUFA methyl esters from fish oil by combining ILs and silver salts was first reported by Li and Li (2008). In their work hydrophobic ILs with large anions of low lattice energy were identified as the best. For the case of ester synthesis via direct esterification, lipase/esterase‐mediated catalysis, and using functionalized ionic liquids as dual solvent–catalysts, could be considered a green approach in terms of catalysts and green solvents (Tao et al., 2016). The main advantages of lipase/esterase‐mediated esterification in nonaqueous systems compared to acid/base catalysis include the mild reaction conditions, high selectivity, and creation of less waste (Brígida, Amaral, Coelho, & Goncalves, 2014; Reetz, 2013). Specifically, ILs containing aromatic rings, i.e., the imidazole and pyridinium types, have been reported capable of selectively extracting the healthful *n*‐3 PUFA, thus subsequently enhancing their purity, by forming reversible π–bonding that governs the selective adsorption of PUFA and ethyl esters from fish oil (Cheong et al., 2011; Ventura et al., 2017). Nevertheless, there remains a void in the literature on how such a green system could be applicable in the production of fish oil containing high omega‐3 triglyceride and the stability of resulting products.

In the present study, imidazolium‐based ILs containing different anions were employed to conduct enzymatic catalysis of esterification in the production of high omega‐3 triglyceride oil. The thermal stability and oxidative kinetics of the products were investigated to gain insights on how to increase product stability so that they can be further optimized to meet industrial processing, storage, and application needs.

## 2 MATERIALS AND METHODS

### 2.1 Substrates and reagents

Two types of fish oils, namely triglycerides fish oil (19.21% EPA and 10.81% DHA) and ethyl ester fish oil (42.47% EPA and 31.91% DHA), were kindly provided by the Fujian Coland Marine Bio‐engineering Co., Ltd (Fujian, China). Novozyme 435 (*Candida antarctica* lipase immobilized on acrylic resin) and lipozyme TL IM, an inexpensive 1, 3‐position‐specific lipase from *Thermomyces lanuginosus*, were purchased from Novozymes A/S (Bagsvaerd, Denmark). Porcine pancreas lipase (PPL) was purchased from Shanghai Sanjie Co., Ltd (Shanghai, China). Ionic liquids, namely 1‐butyl‐3‐methylimidazolium tetrafluoroborate ([BMIM][BF_4_]), 1‐butyl‐3‐methylimidazolium hexafluorophosphate ([BMIM][PF_6_]), and 1‐butyl‐3‐methylimidazolium bis(trifluoromethanesulfonyl)imide ([BMIM][Tf_2_N]), were purchased from Shanghai Chengjie Chemical Co., Ltd (Shanghai, China). *N*‐octanoic acid and *n*‐hexane (HPLC grade) were purchased, respectively, from Sinopharm Chemical Reagent Co., Ltd. (Shanghai, China) and Shandong Yuwang industrial Co., Ltd (Shandong, China). Thin‐layer chromatography silica gel G (analytical grade) was purchased from Qingdao Marine Chemical Co., Ltd (Shandong, China). Boron trifluoride diethyl ether and carboxymethyl cellulose sodium (analytical grade) were purchased from Aladdin Chemicals Co., Ltd (Shanghai, China).

### 2.2 Transesterification reaction and separation of triglycerides

The following general procedures were employed for the transesterification reaction. First, 5 g of triglyceride fish oil was mixed with 5 g of ethyl fish oil in a double‐beaker system. The temperature was kept constant (52°C) using a controlled water bath. The reaction began with the addition of 3% lipase (0.3 g) to the mixture under sealed and light‐free conditions. After 24 hr, 2.5‐ml mixture of anhydrous ethanol and acetone was added to terminate the reaction. To obtain the mixed fish oil, the reaction product was filtered to remove lipase and evaporated to remove residual organic solvents (Ruzich & Bassi, 2011). The product was then spotted onto a chromatographic plate and chromatographed. The chromatographic silica gel containing the triglyceride fraction was scraped off. The fraction was retained for methyl esterification reaction. At the end of the reactions, all ILs used were separated and recovered by centrifugation based on ILs' immiscible nature with densities drastically higher than other components in the system (Huddleston, Willauer, Swatloski, Visser, & Rogers, 1998; Liang, Zeng, Yao, & Wei, 2012; Sunitha, Kanjilal, Reddy, & Prasad, 2007).

### 2.3 Methyl esterification of fatty acids

For methyl esterification, 10‐ml 0.5 M NaOH methanol solution was added into the triglyceride fish oil sample, and shaken for 20 min in a constant temperature water bath at 65°C until the oil droplet disappeared. After cooling, 10 ml of methanol‐three boron fluoride ether solution (3:1, v/v; used immediately after preparation) was added, and the mixture was shaken in a constant temperature water bath (70°C) for 20 min. After cooling, 10 ml of *n*‐hexane was added to the mixture and shaken sufficiently for extraction. Saturated salt water was then added to precipitate the oil sample dissolved in *n*‐hexane. Next, the supernatant was transferred into a centrifuge tube, and *n*‐hexane was evaporated under nitrogen flush. The sample was then injected into gas chromatography (see below) for analysis.

### 2.4 Determination of lipase transesterification activity

Five g of *n*‐octanoic acid and 5 g of triglycerides were added into a 25‐ml conical flask with stopper. After stirring uniformly, 1 ml of ionic liquid and 0.1 g of lipase were added under oscillation at 60°C for 2 hr. Approximately 0.2 g of the reaction mixture was transferred immediately to dissolve in 5 ml of *n*‐hexane before 1 M KOH solution was added to remove free fatty acids. After methyl esterification, the relative percentage of *n*‐octanoic acid in glycerides was analyzed by gas chromatography (Goujard, Ferre, Gil, Ruaudel, & Farnet, 2009). One transesterification enzyme unit is defined by the amount of enzyme required to transfer 1 μmol of *n*‐octanoic acid to glyceride per min (Janssen, Oosten, Paul, Arends, & Hollmann, 2014).

### 2.5 Thermal analysis

Thermal analysis of fish oil samples was carried out by synchronous thermal analyzer (STA 449F3; Netzsch Co., Germany). Approximately 6 mg of the sample was placed in a ceramic crucible, capped, and subjected to a thermal reaction in air atmosphere under ‘sample + calibration' mode, and the heating rate was set at 5, 10, 15, and 20°C/min, respectively. At each heating rate, an empty crucible was used as a control group, and scanned it as a baseline in the corresponding temperature range. The airflow was fixed at 60 ml/min (Tan & Che, 2002).

### 2.6 Gas chromatographic (GC) analysis

Gas chromatographic analysis was performed with an Agilent 7890A Gas Chromatograph equipped with a 112‐88A7 HP‐88 capillary column and an FID detector. The chromatographic conditions were as follows: injection volume 1 μl; column temperature programed to 140°C, hold for 5 min, 4°C/min, 220°C, then hold until the analysis was complete. When the EPA and DHA contents were analyzed, the inlet and detector temperatures were set at 250 and 280°C, respectively. When the *n*‐octanoic acid content was analyzed, the inlet and detector temperatures were set at 240 and 250°C, respectively. Carrier gas flow rate was at 1.0 ml N_2_/min, 35.0 ml H_2_/min, 350 ml air/min, and tail blowing 40 ml N_2_/min; the split ratio was 20:1 when EPA and DHA content was analyzed, and 100:1 when analyzing *n*‐octanoic acid content (Ha, Mai, & Sang, 2007). The fatty acid was quantified by Agilent Chemstation software, and the relative percentage of various fatty acids was determined by area normalization. The tests were carried out in parallel, and all sample measurements were conducted in triplicates.

## 3 RESULTS AND DISCUSSION

### 3.1 Selection of lipase

**Figure 1:**
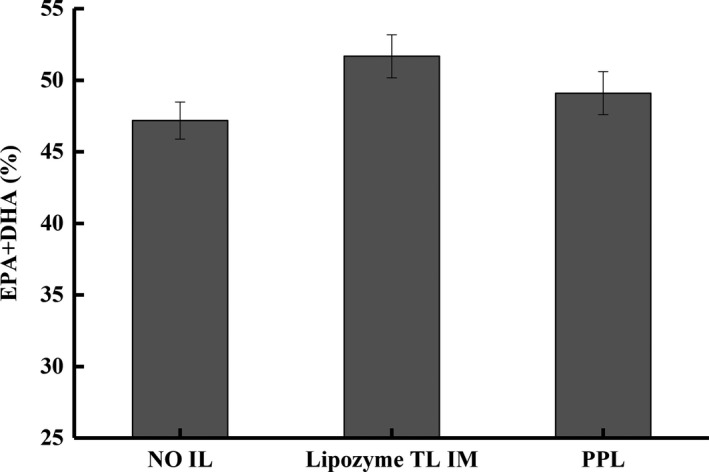
Comparison of the total amount (%) of EPA and DHA attained using Novozyme 345, lipozyme TL IM, and porcine pancreas lipase [PPL] during fish oil transesterification

The effect of different lipases on the transesterification reaction was first assessed to select the most effective enzyme as the catalyst for further studies. Figure 1 shows the total EPA and DHA attained on the esterified triglyceride after equal amount of enzymes was used in the reaction under room temperature without light. Among the three enzymes studied, lipozyme TL IM produced triglyceride with the highest EPA and DHA contents (51.86%), followed by PPL (47.91%), and Novozyme 435 (47.18%). The effect of enzymes on the full composition of fatty acids (saturated, monounsaturated, and polyunsaturated fatty acids) has been reported elsewhere by the authors (Li et al., 2018).

Carvalho, Campos, Noffs, Fregolente, and Fregolente (2009) reported that EPA and DHA as well as other *n*‐3 PUFA tend to attach to stereospecific Sn‐2 position on the glycerol backbone during esterification, whereas low degree of unsaturation or saturated fatty acids usually attach to Sn‐1 and Sn‐3 positions. Therefore, the Sn‐1,3 specific Lipozyme TL IM (Kim, Kim, Lee, Chung, & Ko, 2002) was able to clip off low degree fatty acids from Sn‐1,3 while leaving long‐chain *n*‐3 PUFA attached to Sn‐2 on the glycerol backbone, yielding higher EPA and DHA contents. Additionally, it has been shown that alcoholysis of vegetable oils is faster with Lipozyme TL IM than with Novozyme 435 (Hernández‐Martín & Otero, 2008), indicating that Lipozyme TL IM performs more effectively in hydrolytic reactions than Novozyme 435. A lipase produced by immobilized *Thermomyces (Humicola) lanuginosa*, Lipozyme TL IM has hydrophilic character besides its Sn‐1,3 specificity. It is less expensive than the commonly applied commercial lipase Novozyme 435, thus offering an opportunity for industry to reduce the process cost of high omega‐3 triglyceride oil.

### 3.2 Effect of ILs on transesterification

**Figure 2:**
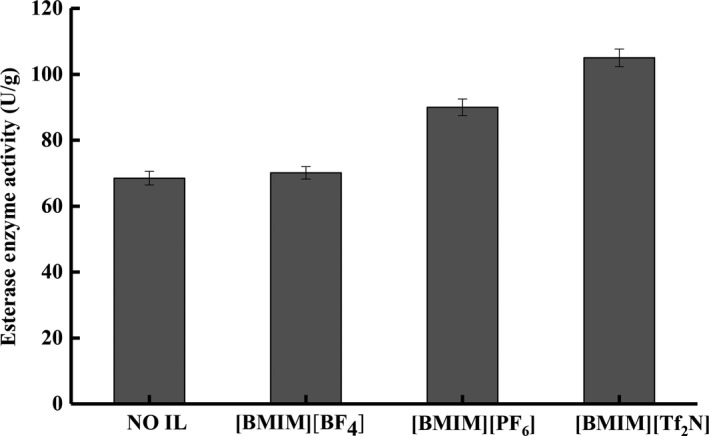
The esterase enzyme activity (U/g) in different ionic liquid (IL) systems [BMIM][BF_4_], [BMIM][PF_6_], and [BMIM][Tf_2_N]

To characterize the effect of ILs on the enzymatic activity during transesterification, *n*‐octanoic acid was employed due to its low cost and low viscosity that favors the enzymatic reactions with triglyceride without needing any solvent. As can be seen in Figure 2, without using any IL, the enzymatic activity of Lipozyme TL IM was 65.75 U/g. The enzymatic activity was elevated with the addition of ILs, with both hydrophobic ILs [BMIM][PF_6_] (90.75 U/g) and [BMIM][Tf_2_N] (99.00 U/g) showing higher enzymatic activity than the hydrophilic [BMIM][BF_4_] (69.75 U/g). For the present work, [BMIM][PF_6_] was chosen as the IL system for further studies, since the cost of [BMIM][Tf_2_N] nearly doubles that of [BMIM][PF_6_] with only incremental increase in the enzymatic activity.

Due to the drastically higher density of ILs compared to fish oil, triglyceride, and the immobilized enzymes used in the present study, the enhanced transesterification could be attributed the phase separation caused by ILs, which significantly increased the surface areas between enzyme and the reactants, resulting in accelerated reaction rate (Nowicki & Muszynski, 2014). According to Ventura et al. (2017), the lipase‐catalyzed transesterification of fish oil could be categorized under liquid–liquid extraction with hydrophobic ILs. Although ILs are still quite pricey at present, hydrophobic ILs ([PF_6_]‐based) have been effectively applied to extract different bioactive compounds (Erbeldinger et al., 2000). It has been reported that the process reached equilibrium relatively quickly and can be further enhanced by increasing the volume ratio of IL to solvent (Zhao et al., 2005). The possibility of recyclability, as well as the simplicity of the procedure resulted in cost efficiency as compared to existing transesterification process. However, the number of available hydrophobic water‐immiscible ILs is much more limited when compared to water‐miscible ones. This suggests certain limitations in terms of variability and tuning of the IL chemical structures aiming at optimizing the performance of such systems.

### 3.3 Effect of ILs on lipase catalyzed fish oil transesterification

**Figure 3:**
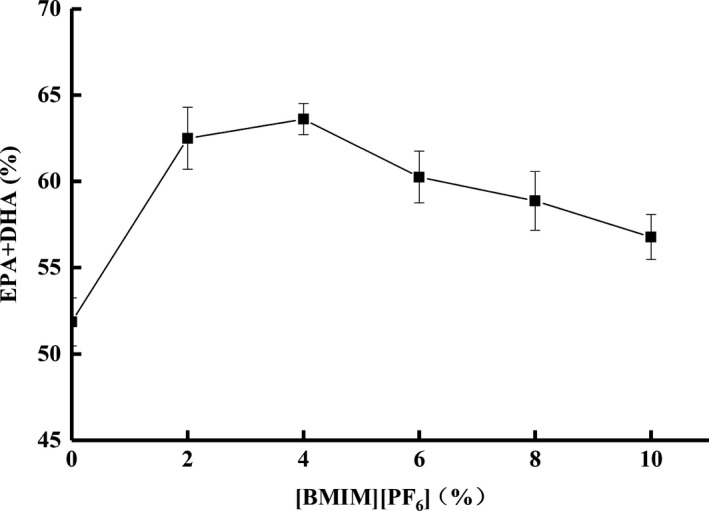
The total amount (%) of EPA and DHA produced using different concentrations (%, w/w) of ionic liquid [BMIM][PF_6_]

When [BMIM][PF_6_] was employed in the mixture of triglyceride‐type and ethyl ester‐type fish oil containing 30.02% and 74.38%, respectively, of omega‐3 fatty acids, the resulting EPA and DHA contents were found to be dependent on the concentration of [BMIM][PF_6_] (Figure 3). Prior to adding any [BMIM][PF_6_], the transesterification reaction yielded 51.86% of total EPA and DHA. The total yield was significantly increased (62.50%) with the addition of 2% [BMIM][PF_6_], and reached 63.60% in the system containing 4% [BMIM][PF_6_]. However, further increase in [BMIM][PF_6_] concentration didn't increase and in fact hindered the total EPA and DHA compared to 4% [BMIM][PF_6_], as can be seen in Figure 3.

As indicated by Vidya and Chadha (2009), [BMIM][PF_6_] could serve as an excellent stabilizer for lipase in the system, which in turn promotes the transesterification reaction. On the other hand, the hydrophobic nature of [BMIM][PF_6_] not only helped retain a water layer around the lipase, but it also provided an adequate microenvironment that reduced the distance between reactants, hence enhancing the activity and stability of the enzyme (Linder, Kochanowski, Fanni, & Parmentier, 2005). However, as the concentration of [BMIM][PF_6_] increased to 6% and beyond, the reaction rate was decreased, which could be attributed to (a) dilution of the reactants in the system that prevented the reaction from going forward, and (b) the increase in system viscosity due to the viscosity of [BMIM][PF_6_], which hindered the transesterification reaction, reducing the total EPA and DHA yield.

### 3.4 Thermal analysis

**Figure 4:**
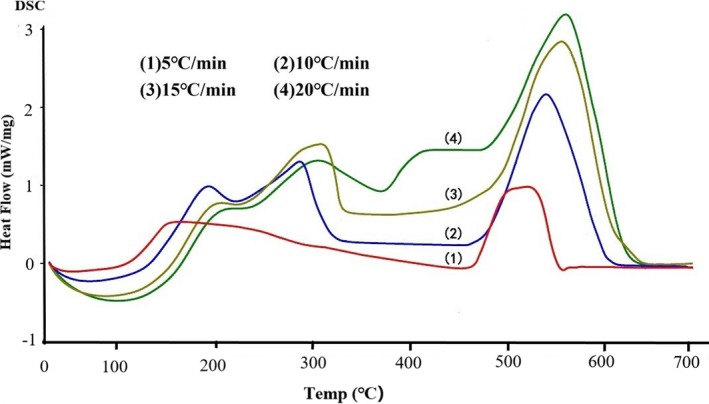
The thermal stability (mW/mg) of the triglyceride‐type fish oil produced in the present study up to 700°C under various temperature gradients (5, 10, 15, and 20°C/min)

**Figure 5:**
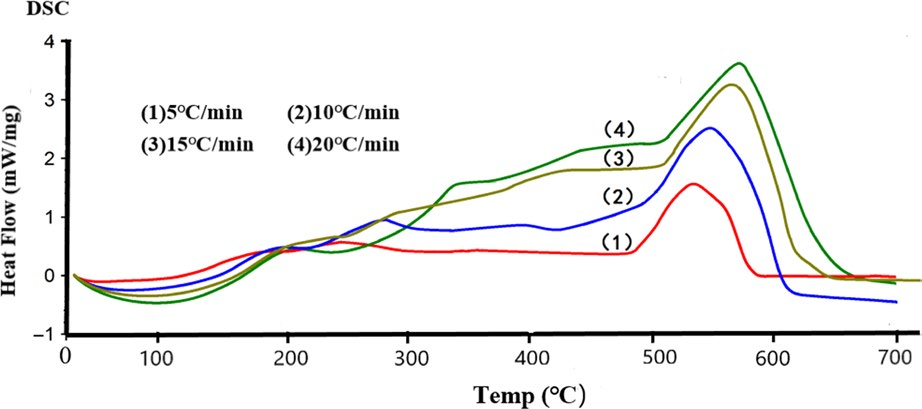
The thermal stability (mW/mg) of a commercial ethyl ester‐type fish oil product containing 40.04% EPA and 20.36% DHA up to 700°C under various temperature gradients (5, 10, 15, and 20°C/min)

The thermal stability of the triglyceride‐type fish oil produced in the present study was examined using DSC (Figure 4) in comparison with a compatible commercial ethyl ester‐type fish oil product containing 40.04% EPA and 20.36% DHA (Figure 5). In general, the DSC curves showed similar trends in Figures 4 and 5, and the peak positions shifted to a higher temperature with increasing temperature. Thermal stability of fish oil is one of the major concerns to the fish oil industry because the inherent oxidation tendency of the high *n*‐3 PUFA contents could result in oxidized products that pose toxic threats to human health, including accelerated lipid oxidation in human body, destruction of cell membrane that consequently deteriorates cell functionality, and cardiovascular diseases (Choe & Min, 2006; Seppanen & Csallany, 2002).

**Figure 6:**
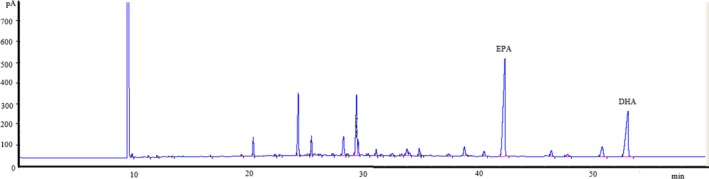
The fatty acid composition of the triglyceride‐type fish oil produced in the present study

**Figure 7:**
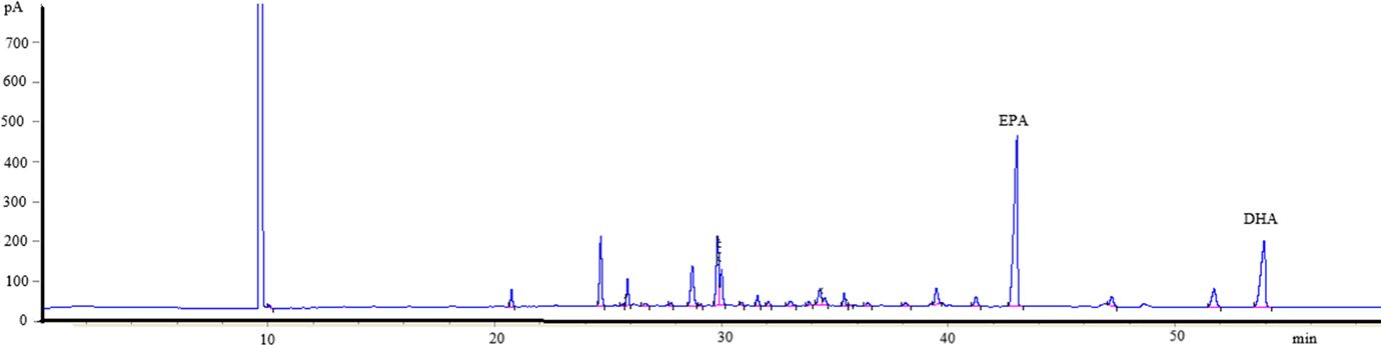
The fatty acid composition of the commercial ethyl ester‐type fish oil employed in the study

Equally noteworthy is that the DSC curves of the triglyceride‐type fish oil (Figure 5) showed more small peaks at lower temperatures (around 200 and 300°C) than that of commercial ethyl ester‐type, indicating that the triglyceride‐type produced in the study contained fatty acids spreading over a wider range of molecular weight than the ethyl ester‐type. This could be attributed to the wide spectrum of acryl groups formed during the transesterification process when producing triglyceride‐type fish oil. Such an observation was further confirmed by analyzing the fatty acid composition of both the triglyceride‐type and the commercial ethyl ester‐type fish oil (Figure 6; 7). As can be seen in Figure 6, the triglyceride‐type fish oil contained 40 fatty acids, whereas the ethyl ester‐type fatty acid contained only 29 fatty acids (Figure 7). Full fatty acid compositions (EPA, DHA and other fatty acids) of these oils have been given in details by the authors previously (Li et al., 2018).

### 3.5 Assessment of kinetic parameters

**Figure 8:**
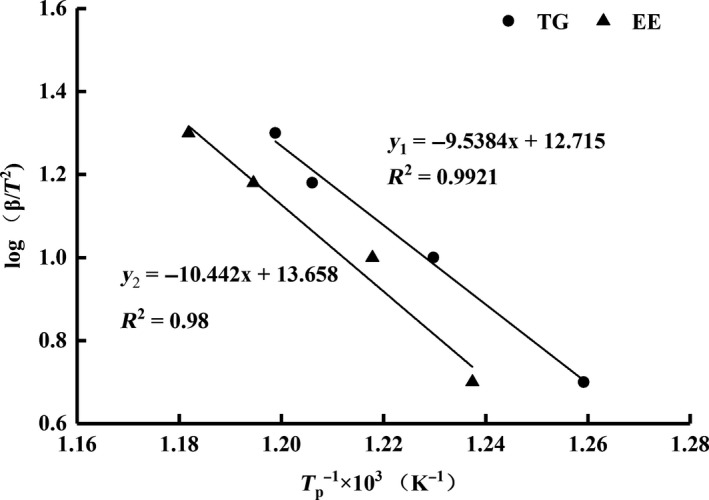
The activation energy (E) of triglyceride‐type and ethyl ester‐type fish oil could be attained from the slop of the regression lines when plotting log β against 1/T based on the Ozawa–Flynn–Wall isoconversional method

To assess the activation energy of the transesterification reaction, as well as to identify critical kinetic parameters, the Ozawa–Flynn–Wall isoconversional method was employed (Mothé & de Miranda, 2013; Ozawa, 1965). The activation energy (E) of triglyceride‐type and ethyl ester‐type fish oil could be attained from the slop of the regression lines when plotting log (β/T^2^) against 1/T (Figure 8). As can be seen in Table 1, the activation energy of commercial ethyl ester‐type fish oil was slightly higher than that of the triglyceride‐type fish oil, indicating that the ethyl ester‐type fish oil had a better stability against thermal oxidation than the triglyceride‐type. However, as the triglyceride‐type fish oil carried a higher degree of unsaturation than the commercial ethyl ester‐type, the difference in activation energy was acceptable, especially when both showed similar kinetic parameters (Table 1).

**Table 1:** Comparison of the thermodynamic characteristics between triglyceride (TG)‐ and ethyl ester (EE)‐type fish oil in terms of their heating rate, peak temperature, and activation energy

Fish oil	Heating rate, (K/min)	Peak temp., Tp (K)	Activation energy, E (KJ/mol)	K (per min)	lnA	*t*_1/2_ (min)
TG	5	794.15	173.64	0.17	24.50	4.19
	10	813.15		0.32	24.53	2.19
	15	829.15		0.46	24.40	1.52
	20	834.15		0.60	24.52	1.15
EE	5	808.15	190.09	0.17	26.55	3.96
	10	821.15		0.34	26.76	2.04
	15	837.15		0.48	26.60	1.42
	20	846.15		0.64	26.57	1.09

## 4 CONCLUSION

This study successfully produced triglyceride‐type fish oil containing elevated concentration of omega‐3 fatty acids by carrying out lipase‐catalyzed transesterification in an IL system. With the presence of 4% [BMIM][PF_6_], Lipozyme TL IM was able to catalyze triglyceride transesterification, reaching the total of 63.60% EPA and DHA content. The thermodynamic characteristics of the triglyceride‐type fish oil were compatible to commercially available ethyl ester‐type, but the former carried more fatty acids than the latter. The kinetic parameters acquired from the present study could serve as the foundation for process scale‐up.

## ETHICAL STATEMENT

There is no conflict of interest to declare. This article does not contain any studies with human participants or animals performed by any of the authors.

## References

[fsn3733-bib-0001] Ackman, R. G. , Ratnayake, W. M. N. , & Olson, B. (1988). The basic fatty acid composition of Atlantic fish oils: Potential similarities useful for enrichment of polyunsaturated fatty acids by urea complexation. Journal of the American Oil Chemists' Society, 65, 136–138. 10.1007/BF02542565

[fsn3733-bib-0002] Baudequin, C. , Baudoux, J. , Levillain, J. , Cahard, D. , Gaumont, A.‐C. , & Plaquevent, J.‐C. (2003). Ionic liquids and chirality: Opportunities and challenges. Tetrahedron: Asymmetry, 14(20), 3081–3093. 10.1016/S0957-4166(03)00596-2

[fsn3733-bib-0003] Brennecke, J. F. , & Maginn, E. J. (2001). Ionic liquids: Innovative fluids for chemical processing. American Institute of Chemical Engineers Journal, 47, 2384–2389. 10.1002/(ISSN)1547-5905

[fsn3733-bib-0004] Brígida, A. I. , Amaral, P. F. , Coelho, M. A. , & Goncalves, L. R. (2014). Lipase from Yarrowia lipolytica: Production, characterization and application as an industrial biocatalyst. Journal of Molecular Catalysis B: Enzymatic, 101, 148–158. 10.1016/j.molcatb.2013.11.016

[fsn3733-bib-0006] Carvalho, P. D. O. , Campos, P. R. , Noffs, M. D. A. , Fregolente, P. B. , & Fregolente, L. V. (2009). Enzymatic hydrolysis of salmon oil by native lipases: Optimization of process parameters. Journal of the Brazilian Chemical Society, 20(1), 117–124. 10.1590/S0103-50532009000100019

[fsn3733-bib-0007] Cheong, L. Z. , Guo, Z. , Yang, Z. , Chua, S. C. , & Xu, X. (2011). Extraction and enrichment of n‐3 polyunsaturated fatty acids and ethyl esters through reversible π–π complexation with aromatic rings containing ionic liquids. Journal of Agricultural and Food Chemistry, 59(16), 8961–8967. 10.1021/jf202043w 21790198

[fsn3733-bib-0008] Choe, E. , & Min, D. B. (2006). Mechanisms and factors for edible oil oxidation. Comprehensive Reviews in Food Science and Food Safety, 5(4), 169–186. 10.1111/j.1541-4337.2006.00009.x

[fsn3733-bib-0009] Cooney, M. J. , & Benjamin, K. (2016). Ionic liquids in lipid extraction and recovery In Ionic liquids in lipid processing and analysis (pp. 279–316). New York City, NY: Elsevier 10.1016/B978-1-63067-047-4.00009-X

[fsn3733-bib-0010] Eltweri, A. M. , Thomas, A. L. , Metcalfe, M. , Calder, P. C. , Dennison, A. R. , & Bowrey, D. J. (2017). Potential applications of fish oils rich in omega‐3 polyunsaturated fatty acids in the management of gastrointestinal cancer. Clinical Nutrition, 36(1), 65–78. 10.1016/j.clnu.2016.01.007 26833289

[fsn3733-bib-0011] Erbeldinger, M. , Mesiano, A. J. , & Russell, A. J. (2000). Enzymatic catalysis of formation of Z‐aspartame in ionic liquid− an alternative to enzymatic catalysis in organic solvents. Biotechnology Progress, 16(6), 1129–1131. 10.1021/bp000094g 11101345

[fsn3733-bib-0012] Goujard, L. , Ferre, E. , Gil, G. , Ruaudel, F. , & Farnet, A. M. (2009). A method to quantify transesterification activities of lipases in litters. Journal of Microbiological Methods, 78(2), 127 10.1016/j.mimet.2009.04.012 19426767

[fsn3733-bib-0013] Ha, S. H. , Mai, N. L. , & Sang, H. L. (2007). Lipase‐catalyzed biodiesel production from soybean oil in ionic liquids. Enzyme and Microbial Technology, 41(4), 480–483. 10.1016/j.enzmictec.2007.03.017

[fsn3733-bib-0014] Halldorsson, A. , Kristinsson, B. , & Haraldsson, G. G. (2004). Lipase selectivity toward fatty acids commonly found in fish oil. European Journal of Lipid Science and Technology, 106(2), 79–87. 10.1002/(ISSN)1438-9312

[fsn3733-bib-0015] He, C. , Li, S. , Liu, H. , Li, K. , & Liu, F. (2005). Extraction of testosterone and epitestosterone in human urine using aqueous two‐phase systems of ionic liquid and salt. Journal of Chromatography A, 1082(2), 143–149. 10.1016/j.chroma.2005.05.065 16035355

[fsn3733-bib-0016] Hernández‐Martín, E. , & Otero, C. (2008). Different enzyme requirements for the synthesis of biodiesel: Novozym^®^ 435 and Lipozyme^®^ TL IM. Bioresource Technology, 99(2), 277–286. 10.1016/j.biortech.2006.12.024 17321130

[fsn3733-bib-0017] Huddleston, J. G. , Visser, A. E. , Reichert, W. M. , Willauer, H. D. , Broker, G. A. , & Rogers, R. D. (2001). Characterization and comparison of hydrophilic and hydrophobic room temperature ionic liquids incorporating the imidazolium cation. Green Chemistry, 3(4), 156–164. 10.1039/b103275p

[fsn3733-bib-0018] Huddleston, J. G. , Willauer, H. D. , Swatloski, R. P. , Visser, A. E. , & Rogers, R. D. (1998). Room temperature ionic liquids as novel media for ‘clean' liquid–liquid extraction. Chemical Communications, 16, 1765–1766. 10.1039/A803999B

[fsn3733-bib-0019] Janssen, L. M. G. , Oosten, R. V. , Paul, C. E. , Arends, I. W. C. E. , & Hollmann, F. (2014). Lipase‐catalyzed transesterification of ethyl formate to octyl formate. Journal of Molecular Catalysis B Enzymatic, 105(7), 7–10. 10.1016/j.molcatb.2014.03.016

[fsn3733-bib-0020] Kim, I. H. , Kim, H. , Lee, K. T. , Chung, S. H. , & Ko, S. N. (2002). Lipase‐catalyzed acidolysis of perilla oil with caprylic acid to produce structured lipids. Journal of the American Oil Chemists' Society, 79(4), 363–367. 10.1007/s11746-002-0489-3

[fsn3733-bib-0023] Li, M. , Fu, H. , Zhang, C. P. , Ouyang, Q. X. , Wang, L. , & Ye, X. Y. (2018). Effects of ionic liquids on omega‐3 fatty acids of fish oil in transesterification products catalyzed various lipase. Journal of Fuzhou University Science Edition, 46(2), 269–274.

[fsn3733-bib-0024] Li, M. , & Li, T. (2008). Enrichment of omega‐3 polyunsaturated fatty acid methyl esters by ionic liquids containing silver salts. Separation Science and Technology, 43(8), 2072–2089. 10.1080/01496390802064174

[fsn3733-bib-0025] Liang, J. , Zeng, W. , Yao, P. , & Wei, Y. (2012). Lipase‐catalyzed regioselective synthesis of palmitolyglucose ester in ionic liquids. Advances in Biological Chemistry, 2(03), 226 10.4236/abc.2012.23027

[fsn3733-bib-0026] Linder, M. , Kochanowski, N. , Fanni, J. , & Parmentier, M. (2005). Response surface optimisation of lipase‐catalysed esterification of glycerol and n‐3 polyunsaturated fatty acids from salmon oil. Process Biochemistry, 40(1), 273–279. 10.1016/j.procbio.2004.01.014

[fsn3733-bib-0027] Lue, M. , Guo, Z. , & Xu, X. (2007). Lipid processing in ionic liquids. Lipid Technology, 19, 204–207. 10.1002/(ISSN)1863-5377

[fsn3733-bib-0028] Mothé, C. G. , & de Miranda, I. C. (2013). Study of kinetic parameters of thermal decomposition of bagasse and sugarcane straw using Friedman and Ozawa–Flynn–Wall isoconversional methods. Journal of Thermal Analysis and Calorimetry, 113(2), 497–505. 10.1007/s10973-013-3163-7

[fsn3733-bib-0029] Nowicki, J. , & Muszynski, M. (2014). Ionic liquids as catalysts and reaction media in oleochemical raw materials processing: A review. Current Organic Chemistry, 18(22), 2797–2807. 10.2174/1385272819666140630170909

[fsn3733-bib-0030] Oishi, K. , Konishi, T. , Hashimoto, C. , Yamamoto, S. , Takahashi, Y. , & Shiina, Y. (2018). Dietary fish oil differentially ameliorates high‐fructose diet‐induced hepatic steatosis and hyperlipidemia in mice depending on time of feeding. The Journal of Nutritional Biochemistry, 52, 45–53. 10.1016/j.jnutbio.2017.09.024 29149647

[fsn3733-bib-0031] Ozawa, T. (1965). A new method of analyzing thermogravimetric data. Bulletin of the Chemical Society of Japan, 38(11), 1881–1886. 10.1246/bcsj.38.1881

[fsn3733-bib-0032] Pei, Y. , Wang, J. , Wu, K. , Xuan, X. , & Lu, X. (2009). Ionic liquid‐based aqueous two‐phase extraction of selected proteins. Separation and Purification Technology, 64(3), 288–295. 10.1016/j.seppur.2008.10.010

[fsn3733-bib-0034] Reetz, M. T. (2013). Biocatalysis in organic chemistry and biotechnology: Past, present, and future. Journal of the American Chemical Society, 135(34), 12480–12496. 10.1021/ja405051f 23930719

[fsn3733-bib-0035] Ruxton, C. H. S. , Reed, S. C. , Simpson, M. J. A. , & Millington, K. J. (2004). The health benefits of omega‐3 polyunsaturated fatty acids: A review of the evidence. Journal of Human Nutrition and Dietetics, 17(5), 449–459. 10.1111/j.1365-277X.2004.00552.x 15357699

[fsn3733-bib-0036] Ruzich, N. I. , & Bassi, A. S. (2011). Proposed kinetic mechanism of biodiesel production through lipase catalysed interesterification with a methyl acetate acyl acceptor and ionic liquid [BMIM][PF_6_] co‐solvent. Canadian Journal of Chemical Engineering, 89(1), 166–170. 10.1002/cjce.20378

[fsn3733-bib-0037] Seppanen, C. M. , & Csallany, A. S. (2002). Formation of 4‐hydroxynonenal, a toxic aldehyde, in soybean oil at frying temperature. Journal of the American Oil Chemists' Society, 79(10), 1033–1038. 10.1007/s11746-002-0598-z

[fsn3733-bib-0039] Sivapragasam, M. , Moniruzzaman, M. , & Goto, M. (2016). Recent advances in exploiting ionic liquids for biomolecules: Solubility, stability and applications. Biotechnology Journal, 11(8), 1000–1013. 27312484 10.1002/biot.201500603

[fsn3733-bib-0040] Subash‐Babu, P. , & Alshatwi, A. A. (2018). Effects of increasing ratios of dietary omega‐6/omega‐3 fatty acids on human monocyte immunomodulation linked with atherosclerosis. Journal of Functional Foods, 41, 258–267.

[fsn3733-bib-0041] Sunitha, S. , Kanjilal, S. , Reddy, P. S. , & Prasad, R. B. N. (2007). Ionic liquids as a reaction medium for lipase‐catalyzed methanolysis of sunflower oil. Biotechnology Letters, 29(12), 1881–1885. 10.1007/s10529-007-9471-x 17634866

[fsn3733-bib-0042] Tan, C. P. , & Che, M. Y. (2002). Differential scanning calorimetric analysis of palm oil, palm oil based products and coconut oil: Effects of scanning rate variation. Food Chemistry, 76(1), 89–102. 10.1016/S0308-8146(01)00241-2

[fsn3733-bib-0043] Tao, Y. , Dong, R. , Pavlidis, I. V. , Chen, B. , & Tan, T. (2016). Using imidazolium‐based ionic liquids as dual solvent‐catalysts for sustainable synthesis of vitamin esters: Inspiration from bio‐and organo‐catalysis. Green Chemistry, 18(5), 1240–1248. 10.1039/C5GC02557E

[fsn3733-bib-0044] Teramoto, M. , Matsuyama, H. , Ohnishi, N. , Uwagawa, S. , & Nakai, K. (1994). Extraction of ethyl and methyl esters of polyunsaturated fatty acids with aqueous silver nitrate solutions. Industrial and Engineering Chemistry Research, 33(2), 341–345. 10.1021/ie00026a026

[fsn3733-bib-0045] Ventura, S. P. , e Silva, F. A. , Quental, M. V. , Mondal, D. , Freire, M. G. , & Coutinho, J. A. (2017). Ionic‐liquid‐mediated extraction and separation processes for bioactive compounds: Past, present, and future trends. Chemical Reviews, 117(10), 6984–7052. 10.1021/acs.chemrev.6b00550 28151648 PMC5447362

[fsn3733-bib-0046] Vidya, P. , & Chadha, A. (2009). The role of different anions in ionic liquids on *Pseudomonas cepacia* lipase catalyzed transesterification and hydrolysis. Journal of Molecular Catalysis B: Enzymatic, 57(1–4), 145–148. 10.1016/j.molcatb.2008.08.007

[fsn3733-bib-0048] Wei, G. T. , Yang, Z. , & Chen, C. J. (2003). Room temperature ionic liquid as a novel medium for liquid/liquid extraction of metal ions. Analytica Chimica Acta, 488(2), 183–192. 10.1016/S0003-2670(03)00660-3

[fsn3733-bib-0049] Yokochi, T. , Usita, M. T. , Kamisaka, Y. , Nakahara, T. , & Suzuki, O. (1990). Increase in the γ‐linolenic acid content by solvent winterization of fungal oil extracted from Mortierella genus. Journal of the American Oil Chemists' Society, 67(11), 846–851. 10.1007/BF02540504

[fsn3733-bib-0050] Zhao, H. , Xia, S. , & Ma, P. (2005). Use of ionic liquids as ‘green'solvents for extractions. Journal of Chemical Technology and Biotechnology, 80(10), 1089–1096. 10.1002/(ISSN)1097-4660

[fsn3733-bib-0051] Zhu, J. , Wang, W. T. , Wang, X. L. , Li, B. , & Wang, Y. Z. (2009). Green synthesis of a novel biodegradable copolymer base on cellulose and poly (p‐dioxanone) in ionic liquid. Carbohydrate Polymers, 76(1), 139–144. 10.1016/j.carbpol.2008.10.004

